# Dysbiosis of intestinal microbiota and decrease in paneth cell antimicrobial peptide level during acute necrotizing pancreatitis in rats

**DOI:** 10.1371/journal.pone.0176583

**Published:** 2017-04-25

**Authors:** Jing Chen, Chunlan Huang, Jingjing Wang, Hui Zhou, Yingying Lu, Lihong Lou, Junyuan Zheng, Ling Tian, Xingpeng Wang, Zhongwei Cao, Yue Zeng

**Affiliations:** 1 Department of Gastroenterology, Shanghai General Hospital, Shanghai Jiao Tong University School of Medicine, Shanghai, China; 2 Shanghai Key Laboratory of Pancreatic Diseases, Shanghai General Hospital, Shanghai Jiao Tong University School of Medicine, Shanghai, China; 3 International Medical Care Center, Shanghai General Hospital, Shanghai Jiao Tong University School of Medicine, Shanghai, China; University of Szeged, HUNGARY

## Abstract

**Objectives:**

Intestinal barrier dysfunction plays an important role in acute necrotizing pancreatitis (ANP) and intestinal microbiota dysbiosis was involved in intestinal barrier failure. Paneth cells protect intestinal barrier and are associated with intestinal microbiota. Here, we investigated changes in intestinal microbiota and antimicrobial peptides of Paneth cells in ileum during ANP.

**Methods:**

Rats with ANP were established by retrograde injection of 3.5% sodium taurocholate into biliopancreatic duct and sacrificed at 24h and 48h, respectively. Injuries of pancreas and distal ileum were evaluated by histopathological score. Intestinal barrier function was assessed by plasma diamine oxidase activity (DAO) and D-lactate. Systemic and intestinal inflammation was evaluated by TNFα, IL-1β and IL-17A concentration by ELISA, respectively. 16S rRNA high throughput sequencing on fecal samples was used to investigate the changes in intestinal microbiota in the ANP group at 48h. Lysozyme and α-defensin5 were measured by real-time PCR, western blot and immunofluoresence.

**Results:**

ANP rats had more severe histopathological injuries in pancreas and distal ileum, injured intestinal barrier and increased expression of TNFα, IL-1β and IL-17A in plasma and distal ileum compared with those of the sham-operated (SO) group. Principal component analysis (PCA) showed structural segregation between the SO and ANP groups. Operational taxonomic unit (OTU) number and ACE index revealed decreased microbiota diversity in the ANP group. Taxonomic analysis showed dysbiosis of intestinal microbiota structure. At phyla level, *Saccharibacteria* and *Tenericutes* decreased significantly. At genus level, *Escherichia-Shigella* and *Phascolarctobacterium* increased significantly, while *Candidatus_Saccharimonas*, *Prevotellaceae_UCG-001*, *Lachnospiraceae_UCG-001*, *Ruminiclostridium_5* and *Ruminococcaceae_UCG-008* decreased significantly. Lysozyme and α-defensin5 mRNA expression levels decreased significantly in ANP group at 48h. Protein expression of lysozyme decreased in ANP groups at 24h and 48h. Meanwhile, the relative abundance of *Escherichia-Shigella* correlated inversely with the decrease in lysozyme.

**Conclusion:**

The disorder in intestinal microbiota and decreases of Paneth cell antimicrobial peptides might participate in the pathogenesis of intestinal barrier dysfunction during ANP.

## Introduction

Acute necrotizing pancreatitis (ANP) is a serious systemic disease with a mortality of 7% to 15% [1]. The intestinal barrier dysfunction plays a pivotal role in the development of ANP. Recently, many animal experiments and clinical studies have reported that ANP is tightly related to intestinal barrier failure [2, 3]. However, the exact underlying pathway has not been fully elucidated.

In acute pancreatitis, the injury in intestinal barrier is now proved to be associated with intestinal microcirculation disturbance, excessive release of inflammatory cytokines, injuries in intestinal epithelium and intestinal microbiota dysbiosis [4]. However, only a few studies have explored the role of intestinal microbiota in ANP. By Polymerase Chain Reaction-Denaturing Gradient Gel Electrophoresis (PCR-DGGE), Tan et al. [5] found reduced bacterial diversity, increased *Enterococcus and Enterobacteriaceae* and decreased *Bifidobacterium* in fecal microbiota, thus confirming the intestinal microbiota dysbiosis in patients with severe acute pancreatitis. In another study, Li et al. discovered bacteria derived from gut in peripheral blood of patients with severe acute pancreatitis, including *Escherichia coli*, *Shigella flexneri*, *Enterobacteriaceae bacterium*, which were linked to the severity of acute pancreatitis [6]. In inflammatory bowel diseases (IBD), the dysbiosis of intestinal microbiota contributes to inflammatory condition and exaggerates immune response [7]. The shift in microbial community structure was reported to induce the expression of TNFα and IL-6 expression in intestine [8] and then exaggerated the intestinal barrier injury in IBD. Acute pancreatitis is characterized by excessive release of inflammatory cytokines in both system and intestine [9].

Paneth cells, located at the crypts of small intestine, play a crucial role in maintaining the normality of the intestinal barrier. They secrete a variety of antimicrobial peptides (AMPs) including α-defensins, Reg3r, lysozyme and contribute to shaping and maintaining the intestinal microbiota [10]. Deficiencies in Paneth cell AMPs were reported to be associated with intestinal barrier failure, leading to the bacterial translocation [11], but Paneth cells have not been thoroughly studied in acute pancreatitis.

In the present study, we aimed to explore alterations in intestinal microbiota structure and Paneth cells in rats with ANP.

## Materials and methods

### Animals

All the animal experimental protocols were approved by the Animal Care and Use Committee of Shanghai Jiaotong University and the experiments were performed in accordance with the guidelines of the committee. All surgeries were performed under 2% sodium pentobarbital anesthesia (0.25mL/100g) to minimize suffering of rats.

Male Sprague-Dawley (SD) rats weighing 120 to 150 gram were purchased from Shanghai SLAC laboratory Animal Co.Ltd (Shanghai, China). All rats were housed at a constant room temperature 24°C with a 12-hour shift of the light-dark cycle and had free access to water and rat chow. Every five rats were housed in a cage.

Forty SD rats were randomly divided into a sham-operated group (SO group) and a ANP group with 20 rats in each group. Each group was further divided into 24h and 48h subgroups, respectively. After induction of anesthesia, laparotomy was performed through a 2-cm-long midline incision. After identification of the duodenal papilla inside the duodenum duct wall, a No.5 needle was inserted into the biliopancreatic duct. To prevent backflow, two microvascular clamps were temporarily used to nip the end of the duct and occlude the common hepatic duct at the confluence of hepatic duct. After connecting the needle with the transfusion converter, ANP was induced by retrograde injection of 3.5% sodium taurocholate solution at a volume of 0.1mL/100g into the biliopancreatic duct via the microinjection pump at the speed of 0.2mL/min (per rat 200–250g)[12]. The SO group rats underwent a sham operation and were injected with normal saline (0.1mL/100g).Rats were euthanasia by an intraperitoneal injection with the pentobarbital and were sacrificed by exsanguinations under deep anesthesia at 24h and 48h after ANP induction. The dose of euthanasia is about 3 times as much as the amount of narcotics. Blood samples were collected from abdominal aorta. The pancreas and the distal ileum were promptly fixed in 10% neutral buffered formaldehyde solution for further histological examination. Segments of the distal ileum were isolated, washed with cold phosphate-buffered saline, snap-frozen in liquid nitrogen and stored at -80°C for further experiments. Fresh feces of rats in the SO48h and the ANP48h groups were collected in sterile tubes before the rats were sacrificed and stored at -80°C until further analysis.

### Histological examination

For light microscope observation, the pancreatic and ileal tissues fixed in 10% neutral buffered formaldehyde solution were dehydrated, embedded in paraffin and then cut into 4-um sections. The sections were routinely stained with hematoxylin and eosin, and observed by two pathologists who were blinded to the experiment via a light microscope. The morphologic changes of the pancreas, including pancreatic edema, acinar cell necrosis, adipose necrosis, hemorrhage and inflammation, were scored according to the criteria of Schmidt [13]. The pathological damage of the ileum was graded according to the Chiu’s standard [14].

### Analysis of plasma D-lactate and diamine oxidase (DAO)

DAO activity was measured by a commercial kit (Nanjing Jiancheng Bioengineering Institue, China) according to the manufacturer’s instruction. Plasma D-lactate level was determined by using a commercial kit (abcam, UK) in accordance with the manufacturer’s protocol.

### Analysis of TNFα, IL-1β and IL-17A levels in plasma and the distal ileum

The expression levels of inflammatory cytokines such as TNFα, IL-1β and IL-17A in systemic circulation and small intestine were measured by analyzing the plasma and the distal ileum tissue respectively. 1-cm-long segment of the distal ileum was removed, cleaned, snap-frozen in liquid nitrogen and stored at -80°C. Tissues with equal weight (100mg) from each group were homogenized with 1mL PBS containing 1% protease inhibitor. The homogenates were centrifuged at 12000 rpm at 4°C for 5minutes. The supernatants were collected in sterile tubes and stored in -80°C. The concentration of total protein was detected using BCA protein assay kit (Beyotime, China). TNFα, IL-1β and IL-17A expression levels in the plasma and the distal ileum tissues were measured by enzyme-linked immunosorbent assay (ELISA) (eBioscience, USA) according to the manufacturer’s protocols[15].

### DNA extraction, PCR amplification, pyrosequencing and bioinformatics analysis

Bacterial DNA was extracted from 200mg rat feces per sample with the E.Z.N.A. Soil DNA Kit (Omega Bio-Tek, Norcross, GA, USA) according to the manufacturer’s protocols. The concentration of DNA was measured using a NanoDrop2000 (Thermo Scientific, Waltham, MA, USA). Bacterial DNA was amplified with 338F(ACTCCACGGGAGGCA) and 806R(GGACTACHVGGGTWTCT) primers covering V3-V4 region of the bacterial 16S rRNA gene. PCR amplification was conducted in a 20uL reaction volume including 10ng of DNA template, 4μL of FastPfu Buffer, 2μL of dNTPs (2.5mM), 0.8μL of each primer (5μM) (Sangon Biotech, Shanghai, China), 0.4μL of FastPfu Polymerase (TransStart® FastPfu DNA Polymerase, TransGenBioTech, Beijing, AP221-01). The PCR amplified products were checked using 2% (wt/vol) agarose gel electrophoresis and purified using the AxyPrep DNA Gel Extraction Kit (Axygen Biosciences, Union City, CA, USA) according to the manufacturer’s introductions and quantified via QuantiFluor-ST (Promega, Madison, WI, USA). Equimolar concentrations of amplifications were pooled and sequenced on an IlluminaMiSeq platform according to the manufacturer’s instructions.

The raw prosequencing reads were denoised and quality-filtered using Trimmomatic and FLASH software. The high-quality sequences were assigned to samples according to barcodes. Operational taxonomic units(OTUs) were clustered at 97% nucleotide similarity level using UPARSE (version7.1http://drive5.com/uparse/) and chimeric sequences were identified and removed using UCHIME. The taxonomy of each 16S rRNA gene sequence was analyzed by RDP Classifier (http://rdp.cme.msu.edu/) against the SILVA 119 16S rRNA database using a confidence threshold of 70%. OTUs that reached 97% similarity were used for α-diversity estimators, including OTU number and ACE index, and Good’s coverage and rarefaction curve analysis by using Mothur (Version 1.30.2;www.mothur.org/). The PCA was generated in accordance with the Bray-Curtis distance matrix calculated using OTU information from each sample.

### RNA isolation and real-time PCR

Total RNA was isolated from segments of the distal ileum using TRIzol kit (Invitrogen, USA). Reverse transcription (RT) was conducted using the PrimeScript^TM^ RT Master Mix kit (TaKaRa, Shige, Japan). The real-time PCR analysis of lysozyme and α-defensin5 expression was performed via SYBR Premix Ex Taq^TM^ kit (TaKaRa, Shiga, Japan) following the manufacturer’s instructions. The RT reaction was set at 37°C for 15 minutes and then inactivated at 85°C for 5 seconds. The real-time PCR protocol was as follows: denaturation at 95°C for 10 seconds, annealing at 62°C for 30 seconds and extension at 72°C for 30 seconds. Samples were run for 40 cycles. Relative gene expression values were analyzed with 2^-ΔΔCt^ method [16]. All primers were synthesized by Sangon (Shanghai,China). The sequences of primers were as follows: forward 5’AGGAATGGGATGTCTGGCTAC3’ and reverse 5’GGTATCCCACAGGCGTTCTT3’ for lysozyme, forward 5’TCCAGCGCATGAAGACACTT3’ and reverse 5’GTCTCAGCGGCAACAGAGTA3’ for α-defensin5. β-actin was used as an internal control: forward 5’AGGATGCAGAAGGAGATTACTGC3’ and reverse 5’AAACGCAGCTCAGTAACAGTGC3’. All experiments were performed in triplicate.

### Immunofluorescence

Immunofluorescence analysis was performed to observe the protein expression of lysozyme in Paneth cells. Paraffin-embedded sections of the distal ileum were deparaffinized and rehydrated, and then antigen retrieval was applied utilizing citrate buffer. Endogenous peroxidase activity was blocked using hydrogen peroxide (3% in methanol) for 15minutes, and then the slides were incubated with primary antibody at 4°C overnight. The antibody used for single staining was anti-lysozyme (1:1000 dilution, Dako, Denmark). After washing three times with PBS, the sections were incubated with fluorescein-labeled secondary antibody for 30minutes. DNA was stained with DAPI for 5minutes to visualize nucleus.

### Western blot analysis

Western blot analysis was performed to measure the protein expression of lysozyme. Frozen segments of the distal ileum were homogenized in ice-cold RIPA lysis buffer with 1% protease inhibitor. The homogenates were then centrifuged at 12000rpm for 10 minutes at 4°C and the supernatants were collected to measure lysozyme expression. The protein concentrations were measured via a BCA assay kit (Beyotime Biotechnology, China). The extracts were heated at 100°C for 10 minutes and then equal amounts of protein were separated by 15% SDS-PAGE, and then transferred to a PVDF membrane. The membrane was blocked with 5% fat-free milk for 60 minutes and incubated with antibodies against lysozyme (1:1000 dilution, Dako, Denmark) and β-actin (1:1000 dilution, Beyotime, China) overnight at 4°C. The membrane was then washed with Tris Buffered Saline with Tween-20 (TBST) three times and incubated with secondary goat anti-rabbit antibody for 90 minutes at room temperature. Protein bands were visualized by enhanced chemiluminescence (Pierce, Rockford, IL).

### Statistical analysis

Data were presented as mean ± standard deviation (SD). Student’s t-test, Mann-Whitney test and Spearman test were performed using SPSS19.0 software and a *p* value of <0.05 was considered statistically significant.

## Results

### Pathological changes in the pancreas and the distal ileum in rats with ANP

**Fig 1:**
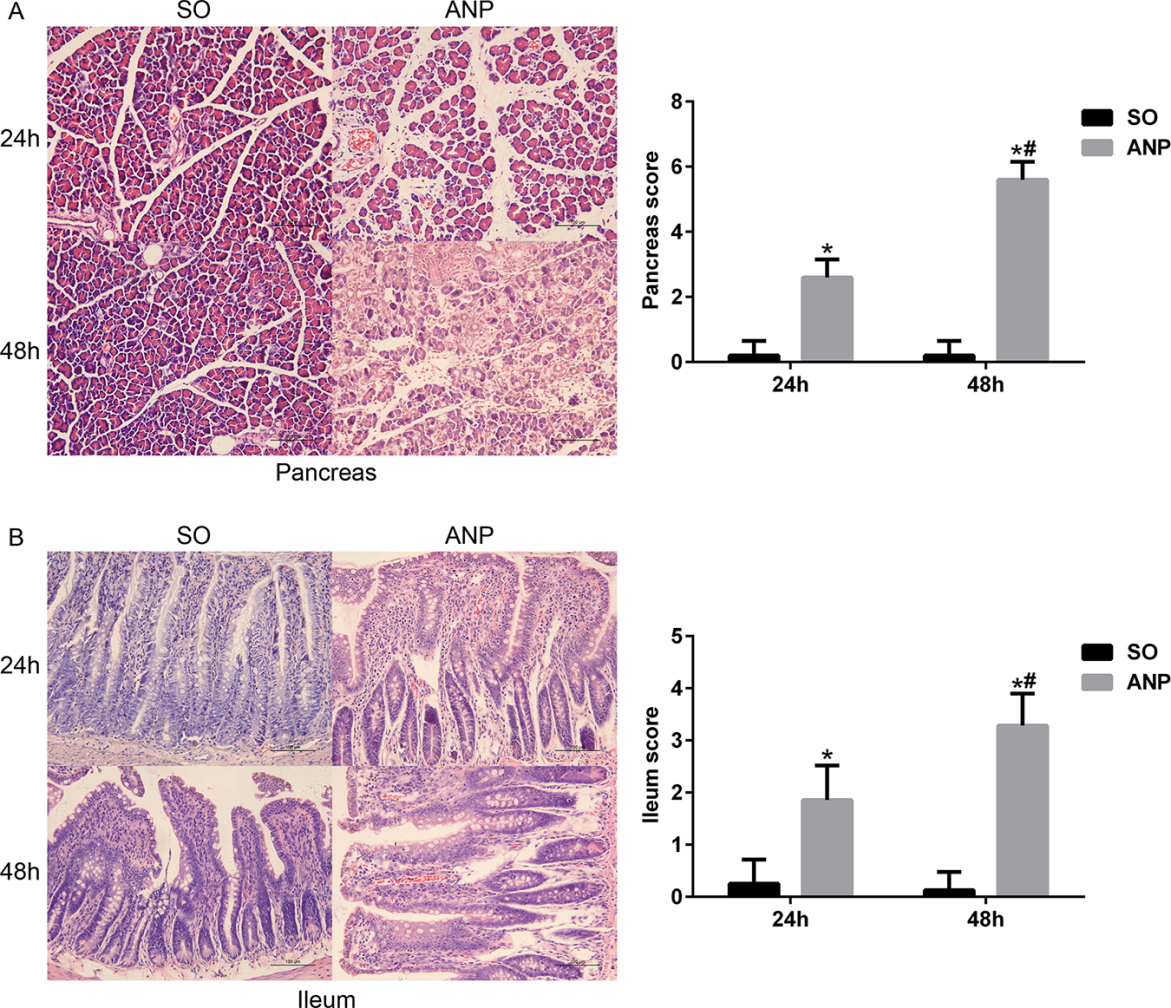
Pathological changes in the pancreas and the distal ileum in rats with ANP. (A) The histopathological changes and the pathological scores of the pancreas of rats (HE, ×200). (B) The histopathological changes and the pathological scores of the distal ileum of rats (HE, ×200). *vs*.SO*p<0.05, *vs*.ANP24h^#^*p*<0,05.

As shown in Fig 1A, in all ANP rats, the pancreatic injury was featured by extensive enlarged interlobular interspaces, patchy necrosis, hemorrhage and inflammatory cell infiltration under light microscope. Compared with the SO groups, the ANP groups had significantly higher histopathological scores at 24h and 48h (*p*<0.05, respectively).The ANP48h group had significantly higher histopathological scores than that of the ANP24h group (*p*<0.05). In Fig 1B, we also found histopathological changes in distal ileum including shortened villi, edema and infiltration of inflammatory cells. In consistence with the severity of pancreas, the distal ileum in the ANP groups had higher pathological scores at 24h and 48h (*p*<0.05, respectively) compared with that of the SO groups. The ANP48h group had significantly higher pathological scores than that of the ANP24h group (*p*<0.05). These results indicated that ileum was injured during ANP. The morphological injury of the pancreas and distal ileum was the most severe at 48h time point after surgery.

### Changes in intestinal barrier permeability and the expression of inflammatory cytokines in the plasma and the distal ileum during ANP

**Fig 2:**
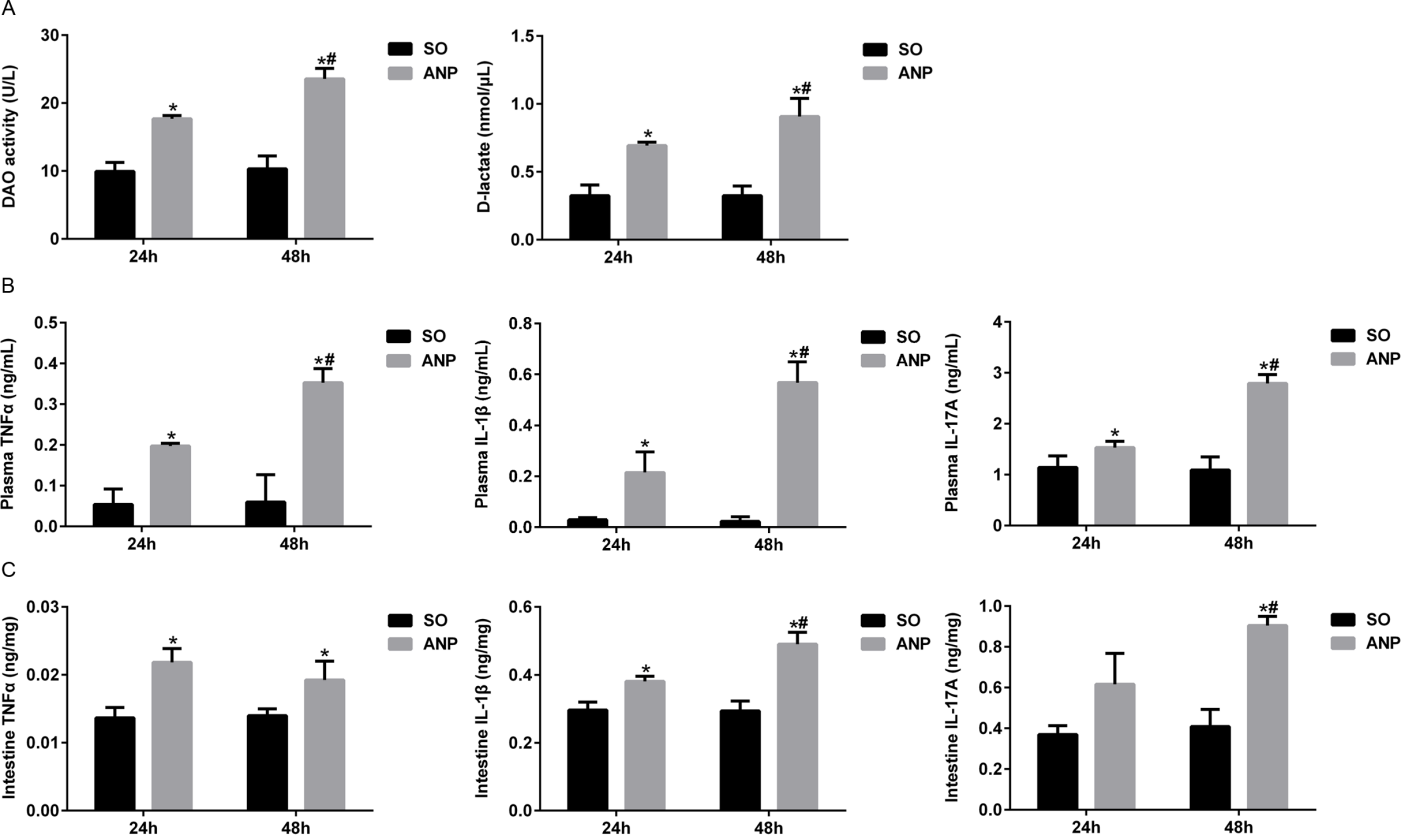
Changes in intestinal barrier permeability and the expression of inflammatory cytokines in the plasma and the distal ileum during ANP. (A) Measurement of plasma diamine oxidase (DAO) activity and D-lactate at 24h and 48h after ANP induction. (B) Plasma inflammatory cytokines TNFα, IL-1β and IL-17A levels in rats at 24h and 48h after ANP induction. (C) Intestinal inflammatory cytokines TNFα, IL-1β and IL-17A at 24h and 48h after ANP induction. *vs*.SO*p<0.05, *vs*.ANP24h^#^*p*<0,05.

To evaluate the severity of intestinal barrier dysfunction, plasma DAO and D-lactate were measured as indicators of intestinal mucosal mass and integrity which can reflect the extent of permeability and damage in the intestine. As shown in Fig 2A, compared with the SO groups, plasma DAO and D-lactate both significantly increased in the ANP groups at 24h and 48h (all *p*<0.05). The ANP48h group had significantly higher levels of plasma DAO and D-lactate than those of the ANP24h group (*p*<0.05, respectively).

TNFα is a key role in severe acute pancreatitis, triggers expression of other inflammatory cytokines such as IL-1β and aggravates the tissue injury. IL-1β is required for the production of IL-17[17]. They are all important proinflammatory cytokines in acute pancreatitis. We evaluated systemic and intestinal inflammation by measuring TNFα, IL-1β and IL-17A levels in plasma and distal ileum respectively. As shown in Fig 2B, there was significant upregulation of plasma TNFα, IL-1β and IL-17A levels in the ANP24h and ANP48h groups (all *p*<0.05). Compared with those of the ANP24h group, the ANP48h group had significantly higher levels of plasma TNFα, IL-1β and IL-17A (all *p*<0.05). We also evaluated the expression of inflammatory cytokines in distal ileum by ELISA. In consistence with the systemic alterations, intestinal TNFα, IL-1β and IL-17A expression in the ANP groups also increased (Fig 2C). Compared with that of the SO groups, TNFα level in ANP rats increased significantly at 24h and 48h (both *p*<0.05). IL-1β level also significantly increased in ANP rats at 24h and 48h compared with that in the SO groups (both *p*<0.05) and had higher level at 48h than that at 24h (*p*<0.05). The intestinal IL-17A expression in the ANP48h group significantly increased compared with that in the SO group and the ANP24h group (*p*<0.05, respectively). All above these indicated that intestinal barrier dysfunction happened in ANP and rats at 48h after ANP induction had the most severe injury in intestine.

### Changes in intestinal microbiota diversity and structure in ANP

**Fig 3:**
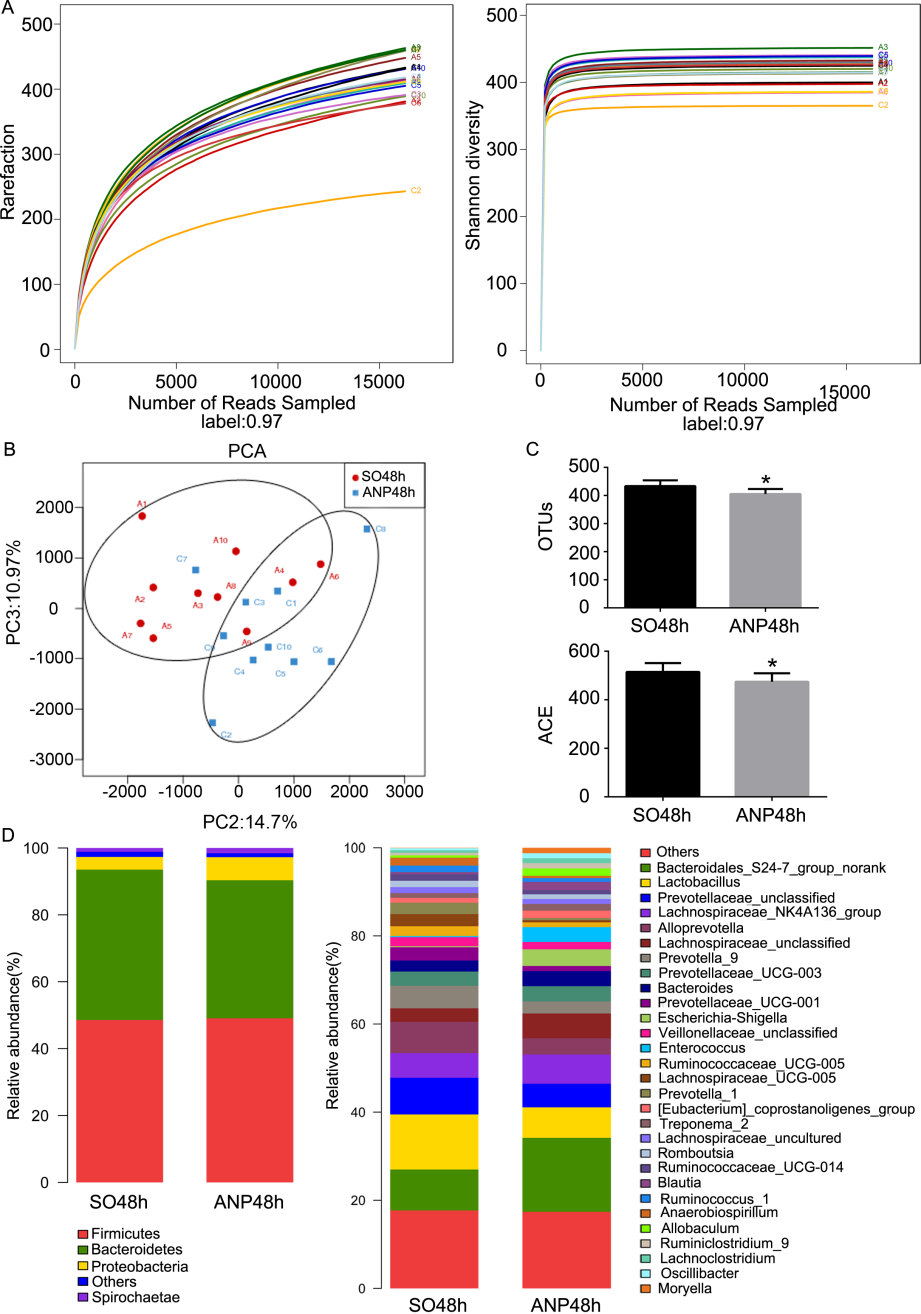
Changes in intestinal microbiota diversity and structure in ANP. (A) Rarefaction curve and Shannon diversity curve of single sample in each group. (B) β-diversity comparison via PCA showed a separation between the SO48h and ANP48h groups. Principle components (PCs) 2 and 3 explained 14.7% and 10.97% of the variance respectively. Each symbol represents one sample. (C) The estimators of intestinal bacterial α-diversity in the SO48h and ANP48h groups. (D) Relative abundance of phyla and genera in intestinal microbiota of the two groups.

A total of 325300 high-quality sequences and 899 OTUs were obtained, using pyrosequencing, from the twenty samples of the SO48h and ANP48h groups. The rarefaction curves tend to approach the saturation plateau and the Shannon diversity of all samples was stable, suggesting that most diversity had already been discovered (Fig 3A). The Good’s coverage index revealed that 99% of the species were obtained in the twenty samples. As shown in Fig 3B, PCA reflected the β diversity by revealing a clear separation between the SO48h and ANP48h groups. It indicated that the overall microbiota structure was different between the two groups. PCA showed that the PC2 and PC3 respectively accounted for 14.7% and 10.97% of the variance and PC2 clearly separated the samples. This result implied the influence that ANP had on gut microbiota structure. OTU number and ACE index, the estimators of community α diversity, reflected lower diversity of gut microbiota in the ANP48h group compared with that in the SO48h group (*p*<0.05, respectively) (Fig 3C).

**Fig 4:**
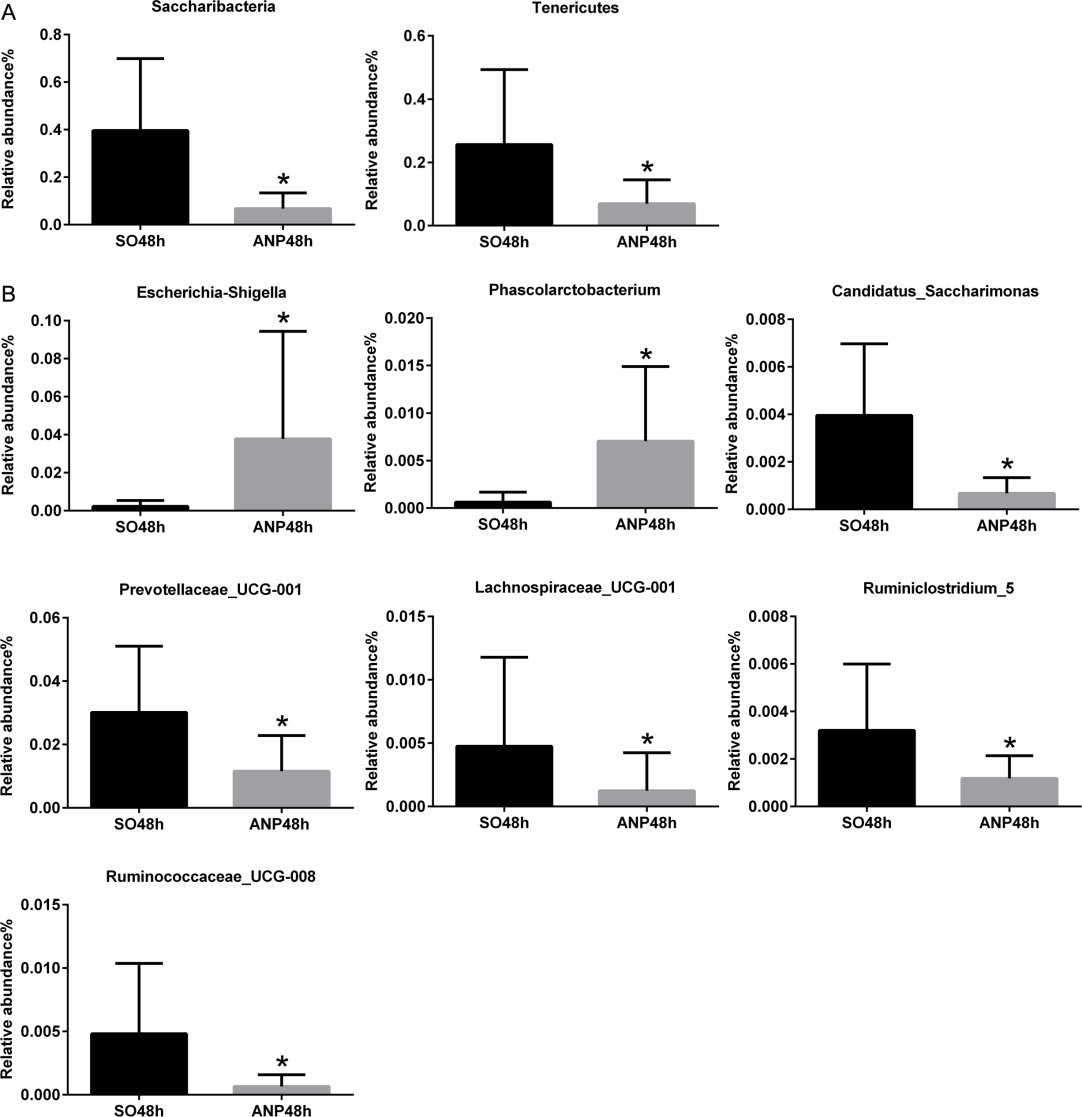
Relative abundance of significantly different phyla and genera betweenthe SO48h and ANP48h groups. (A) The significantly different phyla between the two groups. (B) The significantly different genera between the two groups. *p<0.05

In the phyla level, the ANP48h group had significant lower abundance of *Saccharibacteria* and *Tenericutes*than that of the SO48h group (*p*<0.05, respectively) (Fig 4A). However, none of the two groups had obvious changes in *Firmicutes* and *Bacteroidetes* abundance (Fig 3D). In the genus level, the ANP48h group had higher abundance of *Escherichia-Shigella* and *Phascolarctobacterium* (*p*<0.05, respectively). The abundance of *Candidatus_Saccharimonas*, *Prevotellaceae_UCG-001*, *Lachnospiraceae_UCG-001*, *Ruminiclostridium_5* and *Ruminococcaceae_UCG-008* in the ANP48h group was significantly lower than that in the SO48h group (all *p*<0.05) (Fig 4B). All above these suggested that ANP might play a vital role in leading to the gut microbial structure changes.

### Antimicrobial peptides expression decreased in ANP

**Fig 5:**
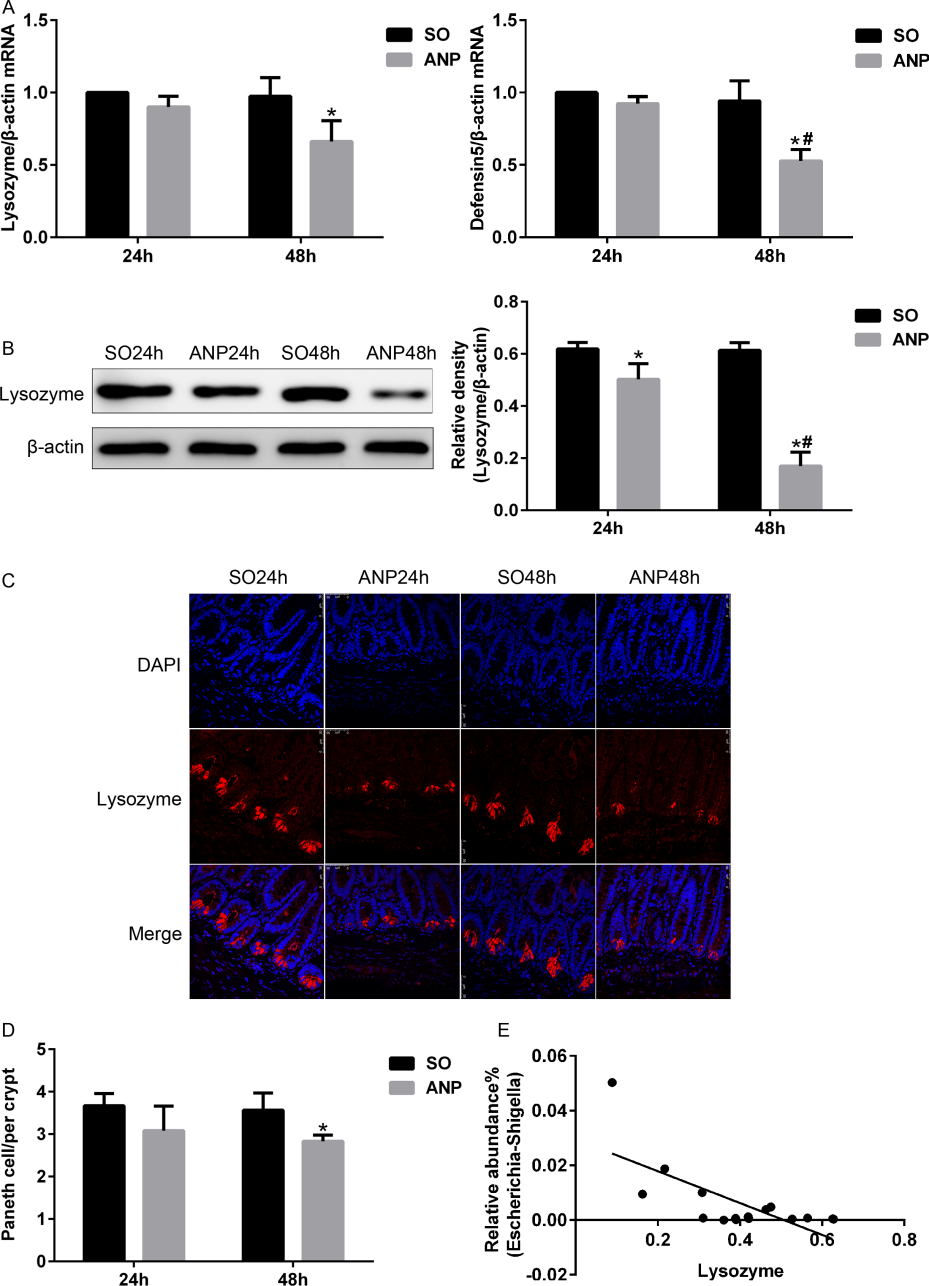
Antimicrobial peptides expression decreased in ANP. (A) Antimicrobial peptides (lysozyme and α-defensin5) mRNA expression by real-time PCR in the distal ileum at 24h and 48h after ANP induction. (B) Lysozyme protein expression by western blot in the distal ileum at 24h and 48h after ANP induction. (C) Lysozyme (red) protein expression produced by Paneth cell in the distal ileum by immunofluorescence. Nuclei were counterstained blue with DAPI. (original magnification, ×200). (D) Quantification of the number of Paneth cells among the SO and ANP groups at 24h and 48h. (E) The relative abundance of *Escherichia-Shigella* correlated inversely with lysozyme protein expression in the SO48h and ANP48h groups. r = -0.57, *p*<0.05. *vs*.SO*p<0.05, *vs*.ANP24h^#^*p*<0.05.

To investigate the alterations in Paneth cells during ANP, real-time PCR, western blot and immunofluorescence were used to measure lysozyme and α-defensin5 in mRNA and protein expression levels. By real-time PCR, lysozyme and α-defensin5 both decreased at 24h and 48h in ANP groups compared with those in the SO groups, and the decrease in the ANP48h group was statistically significant (*p*<0.05) (Fig 5A). By western blot, the ANP groups at 24h and 48h had a significantly decreased level of lysozyme expression compared with that of the SO groups (*p*<0.05). Lysozyme expression further decreased in the ANP48h group compared with that in the ANP24h group (*p*<0.05) (Fig 5B). The immunofluorescence results manifested reduced Paneth cell lysozyme staining in the distal ileum of rats with ANP (Fig 5C).Quantification of Paneth cells revealed that the number of Paneth cells in the ANP48h group decreased significantly compared with that in the SO48h group (Fig 5D). Furthermore, we discovered that the relative abundance of *Escherichia-Shigella* correlated inversely with lysozyme expression by Spearman test (r = -0.57, *p*<0.05) (Fig 5E).

## Discussion

ANP is a severe and emergent disease with multiple complications and a high mortality rate of 7% to 15%[1]. Intestinal barrier dysfunction is now considered as a major reason for the complications such as bacteria translocation and MODS in ANP. According to recent studies, infection of pancreatic and peripancreatic tissues is considered to be originated from intestine [18], especially from the small intestine [19]. In our study, the high pathological scores of intestine and the increased levels of plasma D-lactate and DAO indicated that the intestinal barrier dysfunction became increasingly severe over time after induction of ANP, which was consistent with previous study [20]. After ANP induction, we found intestine had inflammation and the ANP48h group had higher levels of intestinal inflammatory cytokines (TNFα, IL-1β and IL-17A). The excessive release of inflammatory cytokines during acute pancreatitis is a primary reason for intestinal barrier injury. Overproduction of TNFα, IL-1β directly injures intestinal barrier [4]. TNFα, playing a dominating role in severe acute pancreatitis [21], can trigger expression of other inflammatory cytokines such as IL-1β and IL-6 and aggravates the tissue injury [22]. In the present study, the expression level of TNFα increased in both circulation and intestinal tissue, which is in consistence with previous study suggesting that acute pancreatitis was marked with high circulatory concentrations of TNFα [23]. Wang et al. found that expression of TNFα was first up-regulated in the gut among remote organs during severe acute pancreatitis [23]. The causal relationship between intestinal microbiota dysbiosis and excessive inflammation still need further study.

Microbiota in gastrointestinal tract is complex and diverse. The research based on metagenomics has identified 3.3 million microbial genes, up to 10 bacterial phyla and more than 1000 bacterial species in human intestine [24]. Previous studies have suggested that intestinal microbiota disorder stimulated the innate and adaptive immunity and contributed to intestinal barrier dysfunction [25, 26]. In colorectal adenomas, local changed gut environment may favor increased abundance of specific bacterial taxa and some of potential opportunistic pathogens increased, meanwhile, some beneficial bacterial taxa decreased[27]. The shift in structure of intestinal microbiota in turn influenced the inflammatory environment in gut [27]. The exact role intestinal microbiota play in acute pancreatitis has not been clearly illuminated. In the current study, we analyzed the intestinal microbiota structure of the ANP48h group, which had the most significant changes in intestine. Significant shift induced by ANP in the gut microbiota structure was reflected by PCA, and the decreased diversity of gut microbiota was revealed by OTU number and ACE index. Our results showed that at the phyla level, the ANP48h group had decreases in the abundance of *Saccharibacteria* and *Tenericutes*. However, we observed no significant changes in the relative abundance of *Firmicutes* and *Bacteroidetes* which were reported in some studies. At the genus level, there was reduced relative abundance of *Lachnospiraceae_UCG-001*, *Prevotellaceae_UCG-001*, *Ruminiclostridium_5*, *Ruminococcaceae_UCG-008* and *Candidatus_Saccharimonas* in the ANP48h group. In addition, we found increases in relative abundance of *Escherichia-Shigella* and *Phascolarctobacterium* during ANP. All the results collectively suggested an obvious shift in intestinal microbiota structure in ANP. A recent study demonstrated the increased abundance of *Shigella* in blood of patients with acute pancreatitis [6] which is in consistence with our results. Interestingly, Fernandez MI et al.[28] reported that the increased abundance of *Shigella* was induced by disappearance of Paneth cells in Sox9^flox/flox^-vil-cre mice. Paneth cells function as a secondary defensive line of protecting intestinal barrier when the physical intestinal barrier loses [29] and are increasingly identified as guardians of the gut. These highly secretory cells, located at the base of the crypts in small intestine, shape and influence the structure of intestinal bacteria by secreting a variety of AMPs including α-defensins, Reg3r, lysozyme and so on[30]. Lysozyme and α-defensins have the activity against Gram-negative and Gram-positive bacteria while Reg3r only against Gram-positive bacteria [31].

Furthermore, we analyzed the alterations in expression of lysozyme and α-defensin5, two major Paneth cell AMPs. Our results showed that expression of lysozyme in both transcriptional and translational levels obviously reduced after ANP induction. We also found that α-defensin5 at gene level significantly decreased in the ANP48h group. In addition, the number of Paneth cells decreased in the ANP48h group, which may also lead to the reduction of AMP. Interestingly, we found that the significant decrease in AMP correlated inversely with the increased abundance of *Escherichia-Shigella* during ANP. The malfunction of Paneth cells contributes to impairment of intestinal barrier, leading to bacterial translocation and MODS. In a previous research, Teltschik Z et al.[11] discovered the diminished α-defensin5 and 7 expression produced by Paneth cells in rats with cirrhosis, resulting in bacterial translocation. In Paneth cells of starved mice, reduced antimicrobial peptides like lysozyme and cryptdin was observed [32]. Compromised Paneth cells were also present in animal models with intestinal ischemia/reperfusion [33] or acute kidney failure during which Paneth cell disorder was implicated in the occurrence of multiple organ failure [34]. However, at present, there are few researches about Paneth cell alterations in acute pancreatitis. Taken together, these findings demonstrated the abnormality of Paneth cells during ANP and implied that the dysfunction of Paneth cells was involved in intestinal barrier failure in ANP. However, the exact underlying mechanism between Paneth cells and intestinal barrier during ANP need further research.

In summary, the current study showed that ANP rats had aggravated systemic inflammation and intestinal barrier injury. The changes in host, including disorder of intestinal microbiota and the decreased level of AMPs produced by Paneth cells may be one of the mechanisms leading to deterioration of intestinal barrier during ANP. This provides a new insight in exploring the mechanism of ANP.

## Supporting information

S1 File This is excel file containing the analyzed data. (XLSX)
